# Let microorganisms do the talking, let us talk more about microorganisms

**DOI:** 10.1186/s40694-016-0023-9

**Published:** 2016-07-21

**Authors:** Corrado Nai, Boris Magrini, Julia Offe

**Affiliations:** 1 Department Applied and Molecular Microbiology, Institute of Biotechnology, Technical University of Berlin, Gustav-Meyer-Allee 25, 13355 Berlin, Germany; 2 Federation of the European Microbiological Societies (FEMS), Delftechpark 37a, 2628 XJ Delft, The Netherlands; 3 Zurich, Switzerland; 4 Hamburg, Germany

**Keywords:** Microorganisms, Antibiotics crisis, Microbial secondary metabolism, Filamentous fungi, Co-cultivation assays, Science outreach, Science slams, “Art & science”

## Abstract

Microorganisms are of uttermost importance, yet in the eyes of the general public they are often associated with dirt and diseases. At the same time, microbiologists have access to and comprehensive knowledge of just a tiny minority of the microbial diversity existing in nature. In this commentary, we present these issues of public misconception and scientific limitations and their possible consequences, and propose ways to overcome them. A particular interest is directed toward the secondary metabolism of filamentous fungi as well as novel outreach activities, including so-called “science slams” and interactions between the arts and the sciences, to raise awareness about the relevance of microorganisms.

You’ve got to respect microbes. Not because “they are the only culture some people have,” as the comedian Steven Wright puts it. Neither are we talking about a reverential awe, fuelled by recurrent news of killer bugs and pandemic threats. Yes, microbes can be dangerous and can spread diseases easily around the globe. We are occasionally unable to tame them, as recent outbreaks of Ebola or Zika viruses [1, 2] as well as of devastating plant pathogens testify [3]. And despite them being considered the simplest life form on earth, we still don’t know microbes as well as we need to. But in this lies also the beauty of them: in many regards, microorganisms are nature’s treasure trove awaiting to be opened.

The respect we are referring to is related to a fascination for microbes. Studies on microorganisms paved the way for crucial advances in major pillars of our modern society as medicine, human welfare [4–8], industry [9–11] and research [12, 13]. Some environmental species can break down or assimilate toxic compounds or pollutants and are useful in bioremediation [14, 15]. Microorganisms like *Escherichia coli*, *Saccharomyces cerevisiae* and *Neurospora crassa* have been the workhorses of molecular biology and playgrounds for scientific and technological breakthroughs [13, 16, 17]; recent discoveries on the “immune system” of streptococci [18] are currently used as CRISPR/Cas technology to edit genomes across all domains of life, including human zygotes [19] and embryos [20], and fuelling an ongoing scientific revolution [21, 22]. And yet, if at the basis of respect lies understanding, there are still lacunae to overcome—as much for the general public as for biologists or scientists themselves.

## Microbiologists: the hipsters among scientists

**Fig. 1:**
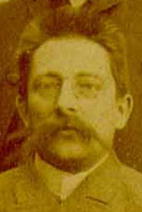
Hipster moustache for hipster microbiologists—here Julius Richard Petri (1852–1921), the German bacteriologist eponymous with the dishes to cultivate microbes. Technical and methodological advances in microbiology have not progressed with the same pace as in other fields of the natural sciences (for details, see text; picture from https://commons.wikimedia.org)

Scientists know and have access to only an estimated 1 % of microbial diversity, as predicted by molecular methods and metagenomics analyses [23]; the rest is referred to as the “microbial dark matter” [24–26]. The main reason is the somehow limited palette of methods at microbiologists’ hand. Since Robert Koch first grew microbial colonies on a potato slice in the late nineteenth century—and soon after on gelatinous, homogeneous media in dishes named after his assistant Julius Petri (Fig. 1)—[27] not much has really changed. Microbiologists still aim for pure cultures of microbes on solid or in liquid media as first step to further analyses, *nolens volens*. They grew microbes before it was cool, and do it today (almost) exactly alike.

These culture-dependent methods are microbiology’s double-edged sword. When they succeed in growing a new strain and in studying it in the laboratory, microbiologists alienate it from its natural habitat: in nature, microbes are highly promiscuous and most likely never grow axenically and in homogeneous substrata. To give a rough idea, a gram of soil harbours an estimate of 10^9^ microbial cells and 1000 different species [28, 29]. The microbial diversity on us (e.g. the skin) and within our body (e.g. the gut) is similarly stunning: microorganisms outnumber our own cells (by a factor of up to ten according to some estimate [30–32]), so that humans are often referred as “superorganisms”. The positive effect of the human microbiome for the health of animals and plants is increasingly acknowledged, even if still poorly understood [33–35]. Similarly, the microbial community context should no longer be overlooked when investigating microbial pathogenesis, a goal that could be achieved by revisiting the classical Koch’s postulates [36].

## Overcoming microbiologists’ culture-dependent limitations

Several protocols successfully overcome some limitations in microbiological methods. It’s now possible to cultivate cells in situ, inoculate them in controlled model systems, recreate natural conditions in the lab as well as grow mixed cultures instead of axenic ones or directly analyse chemical exchanges among microorganisms by mass spectrometry [37–43]. Yet during experiments with standard and reproducible conditions (a prerequisite for scientific consistency) some kind of complexity is invariably lost, and co-cultivation studies with as few as three species are cumbersome and extremely rare [42].

Some interactions amongst microorganisms are known and well characterised, as for example in intra-species communications (e.g. in the processes of quorum sensing [44] or biofilm formation [45, 46]) or in ecologically relevant, positive bipartite partnerships like lichens or mycorrhiza [47–49]. Other examples of associations involving microorganisms are those with marine sponges [50] or within the digestive tract of animals [51]. Due to the complexity and inaccessibility of those microbiomes and habitats, however, the same drawbacks as outlined above exist (in particular, cultivation of the microbes and establishment of model systems to investigate them in the laboratory). For the overwhelming majority of cases, therefore, microorganisms cohabitate the same niche and/or host, and we don’t have any clue which kind of interactions they undergo—like anonymous neighbours in a multistory building who occasionally (if at all) greet each other on the stairway.

## Secondary metabolism with primary significance

Related to this is the fact that, even for well-known industrial organisms studied and exploited for decades like the filamentous ascomycete *Aspergillus niger*, there is a so-called “secondary metabolism” which is mostly inactive under industrially used conditions and axenic growth [52–54]. This metabolism is required for “secondary” roles (e.g. to adapt to stress conditions and the presence of competitors [55, 56]), and it is consequently silent in the controlled settings of a research laboratory or fermentation process. Microbial secondary metabolites with antibiotic, anti-tumoral, psychotropic, cytotoxic, anti-cholesterol or immunosuppressant properties have been isolated [57, 58]. The interest of the microbial secondary metabolism for medicine and biotechnology is undisputed.

Cell-to-cell communication among microbes is investigated as a potent stimulus to activate their silent secondary metabolism [59–64]. When cells are competing with their neighbours for space or resources, they secrete secondary metabolites to engage in a chemical warfare. There seems to be a correlation between microbial promiscuity and ability to secrete secondary metabolites, as exemplified from the fact that soil microorganisms are the primary source of antibiotics [65, 66]. Nonetheless, microorganisms dwelling in scarcely inhabited regions like Antarctica produce antimicrobials as well [67] and these niches should be considered as well when hunting for new putative drug-producing strains.

Antibiotic resistance is on the rise [68–71] and since the last two decades no new class of antimicrobial has been developed [72]. The shadow of a “pre-antibiotic” era does not belong to a distant past but might return as a considerable threat, and new antimicrobials are urgently sought after. Taken together, the need for new antibiotics, compounded by our inability to grow many microorganisms, leads the exploitation of microbial secondary metabolism in natural niches an important experimental goal.

## Filamentous fungi as the workhorses of biotechnology

Among microorganisms, filamentous fungi hold great promises for biotechnology and medicine. In particular, their metabolic and physiological versatility along with their intrinsic resistance toward physical and chemical stresses makes them unequalled among other biotechnologically-relevant microorganisms [73–75]. Recent advances in molecular biology, “-omics” techniques and synthetic biology allowed a better understanding of the molecular cell biology of industrial relevant filamentous fungi, as for example members of the genus *Aspergillus*, making them attractive as microbial cell factories [76]. Having entered the post-genomic era, new exciting possibilities arise for fungal biology [77, 78]. Such knowledge, however, has not been matched by advances in microbiological techniques [27, 79], a reason why the natural way of living of microorganisms is still obscure and this nature’s treasure trove—microbes themselves as well as the secondary metabolism of well-known species—is mostly shut.

We argue that new cultivation techniques are necessary to activate the silent secondary metabolism of filamentous fungi, and, in particular, that co-cultivation experiments under natural conditions (in situ setting) are greatly implementable. One way to do this might be the convergence of existing approaches into a single laboratory tool. The effects of microbial cross-talk in nature might be better exploited by exposing complex microbial communities (e.g. more than 10–100 different species, including unidentified ones) to environmental conditions, instead of grow axenic cultures in nature [38, 39] or co-cultivate two species under laboratory settings [59–64]. This could be a kind of a “black-box” approach with “traceable” and reproducible complexity, in which microbial cross-talk as well as unknown and/or non-standard inputs putatively act in concert to broadly activate microbial secondary metabolism. The platform necessary to achieve this goal seems to be currently missing and needs to be developed and implemented in a suitable laboratory device.

## Microbes as threats, scientists as imaginary characters

Arguably, the general public gropes around in a different kind of darkness than that of microbiologists. As the relevance of (inter-species) microbial cross-talk in nature is still elusive to microbiologists, so seems the communication on the importance of microorganisms to be lagging, at least judging from the public perception of them. Microbes are widely associated with dirt and diseases even though, of all microorganisms, probably just a tiny fraction is pathogenic to humans, animals or plants.

These kind of misconceptions might be due to a general disaffection of society as a whole from scientific facts. Science is complicated and scientific results are occasionally exaggerated, oversimplified or misrepresented by media outlets or universities press-releases [80]. But take a look at popular culture and you won’t find many positive characters representing a realistic scientist either. Subjectively, in the public eye scientists are often seen as either evil Drs. Frankenstein or nerds (without doubting that there might well be even evil nerds among scientists). By the words of comedian John Oliver, science is “the thing we love and respect so much we only allow scientists to be portrayed by the like of Arnold Schwarzenegger, Nicolas Cage and Al Pacino” [80]. Neither is literature scoring better. According to the maxim “write what you know,” it could be a task for scientists to correct that. It is as if, aside from few exceptions [81], established scientists write exclusively memoirs or non-fiction. In line with this, according to the Royal Society in London the best science book ever written is not by a scientist but by the industrial chemist Primo Levi [82], beating among others Charles Darwin, Richard Dawkins and James Watson.

## More than purely interesting

So why should scientists care, and in particular microbiologists? It would be too easy to bluntly put it as Richard Dawkins (who, quoting a journal editor, said “science is interesting and if you don’t agree you can fuck off”) [83] and we won’t. If, as Isaac Asimov believed, “the saddest aspect of life right now is that science gathers knowledge faster than society gathers wisdom,” scientists should feel concerned.

More understanding by the general public in the importance of microorganisms might lead to more acceptance and more public funding to microbial research. Informing the taxpayers on where their money goes and provide an understandable account of the outcomes should also be on the agenda of researchers. To facilitate this, in our opinion the scientific community might consider endeavours to close the gap between science and society as an activity advancing science; science outreaches could be considered as an additional valuable proxy to calculate the impact of researchers.

Misunderstanding about scientific facts can have serious consequences. Amongst the reasons for the increase in antibiotic resistance is the wrong use of these drugs by patients, who for example take them after a common cold or interrupt the treatment after first signs of healing and unnecessarily select for or favour the survival of resistant cells. Of course, several other factors are involved, like the (over-)use of antibiotics in intensive farming [84, 85]; similarly, pharmaceutical companies are less interested in the decade-long development of drugs that easily lose their effectivity [68–71]. Nonetheless, the distance between the general public and science is growing bigger, and ways to close this gap are sought after.

## Science is entertaining, scientists are not nerds

**Fig. 2:**
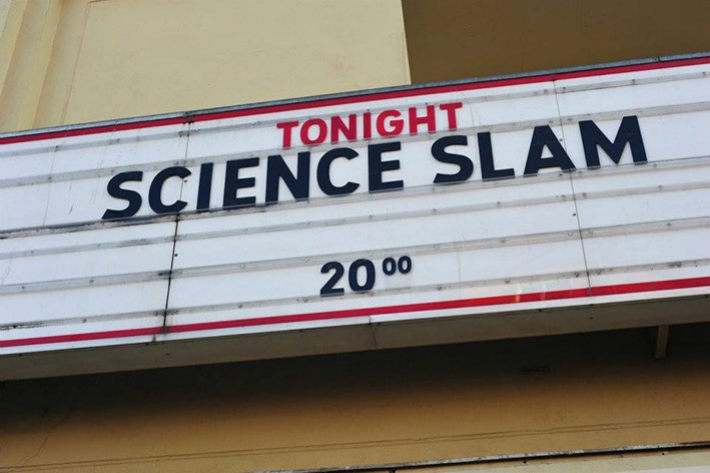
Science slams represent an effective way to close the gap between the scientists and the general audience. The format, which attained much popularity in Germany, foresees that young scientists present their research in an entertaining, easily understandable way (picture by Julia Offe)

One of the new science communication formats that has become increasingly popular is the so-called science slam. In science slams, young scientists explain their own research projects in 10-min talks that are easy to follow, and afterwards the audience gets to vote a winner of the “competition”. The important thing is not primarily the scientific outcome of their work, but to explain it in an understandable, concise and entertaining way. Science slams take place in cultural centers, theaters or clubs, usually in the evening (Fig. 2). So, the scientists leave their ivory tower and become part of popular culture.

The concept, invented and developed in Germany over the past 10 years [86], is now an integral part of science communication in the country, taking place in virtually every university town and regularly attracting a crowd of up to 1500 visitors. The format is spreading throughout other European countries as well as worldwide. The success of science slams is partly due to the fact that it provides the opportunity for direct communication between young scientists and the public. It allows researchers to break from the restraints of classic scientific communication, while the audience experiences their enthusiasm at the forefront of scientific discovery.

Many universities and scientific societies have embraced the science slam as a way to spread knowledge and connect with the public. During this year’s annual conference of the German Association of General and Applied Microbiology, for example, a specific “microbe slam” has been attended by over 1000 microbiologists [87]. Science slams and several other science communication events, like for example the “Long Night of the Sciences” where institutions open their door to the general public, are cementing a collective conscience that science, like sports or the arts, is an integral part of our culture.

## In need of more mixed cultures

Artists can substantially contribute to debates in scientific research. The so-called “microbial art” dates back to one of the founding fathers of microbiology, Alexander Fleming, who used petri dishes as canvas and pigmented cultures as “paint” [88]. Almost a century onward, an increasing number of projects involving artists, curators and institutions have been developed under the common “art & science” label. However, artists working within this field are often pressured to fulfil the role of bridging the gap between the humanities and the sciences. Undeniably, C. P. Snow’s evaluation on the separation between the two cultures has been a recurring reference to emphasize the necessity of collaborative approaches between artists and scientists [89]. We argue that such collaborative work can be achieved in more than one way and can be beneficial to everyone. Artists should feel encouraged to intervene in topical issues by questioning the ethical impacts on the society regarding the implementation of new technologies or by querying the collective understanding of scientific concepts and facts.

**Fig. 3:**
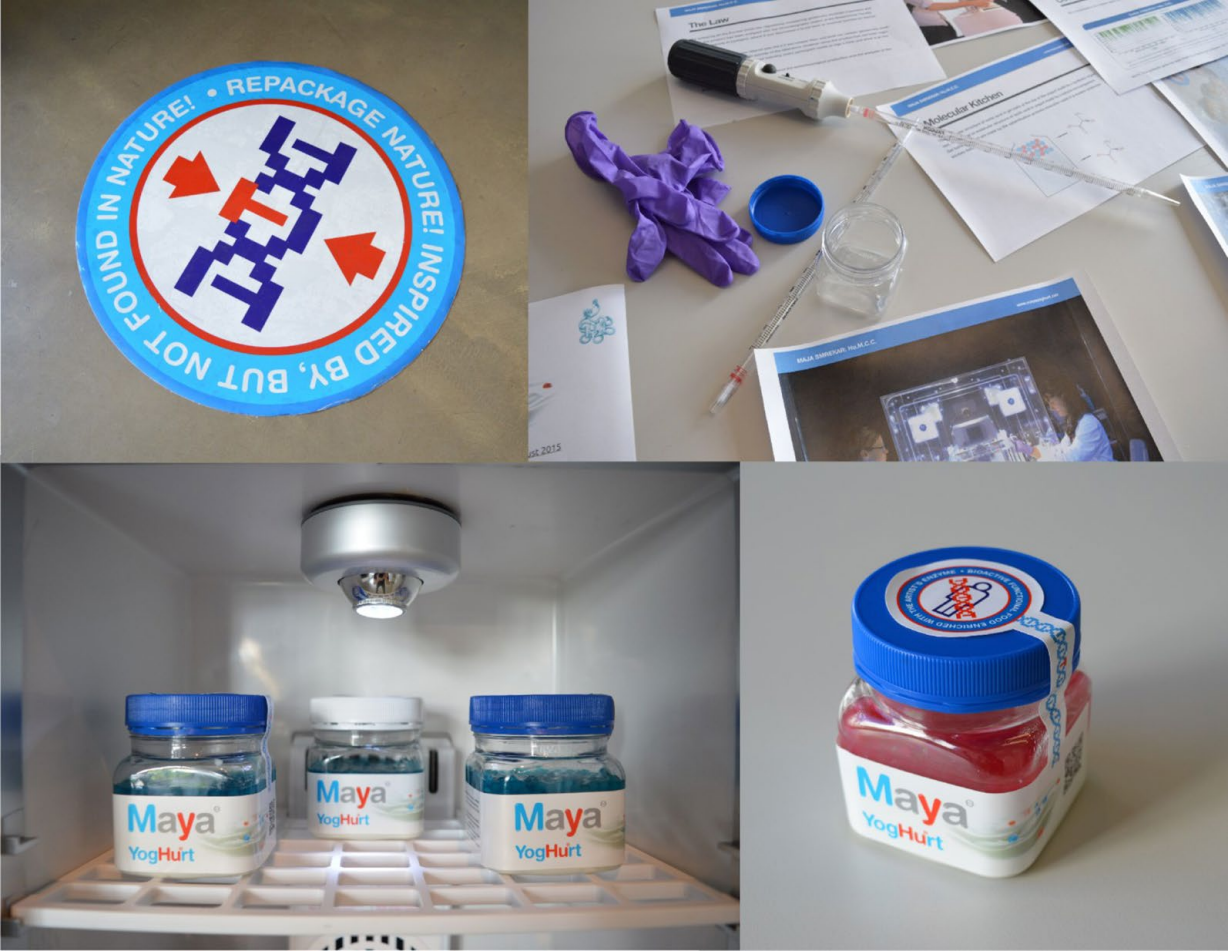
Views of the “Hu.M.C.C.” (for Human Molecular Colonization Capacity) project by Maja Smrekar at the exhibition “The Hydra Project” at Corner College, Zurich (Switzerland) in 2015. Interactions between science and the arts can contribute to the advancement of science and steer it into directions relevant for our modern society (pictures by Boris Magrini)

More specifically, artists have been exploring the possibilities offered by the implementation of biotechnologies. For example, the “Hu.M.C.C.” (for Human Molecular Colonization Capacity) project by Maja Smrekar (Fig. 3) consists of a yogurt produced with a genetically modified microorganism containing the artist’s enzyme [90]. At first glance, the work appears as a moral forewarning about speculative applications of genetic engineering on food production. On the contrary, it is primarily an ironic comment on the increasingly obsessive demand for flawless and wholesome food by consumers and the encouragement to this demand by manufacturer [90]. During the 2016 edition of the Transmediale festival in Berlin, artist François-Joseph Lapointe engaged in 1000 handshakes with the visitors, while regularly collecting and analysing the microbiota of the palm of his hand to generate varying “Microbiome Selfies” [91]. His work is not so much concerned with questions about lack of knowledge on the occurrence of such organisms on our skin, but rather aimed at denouncing the use of antibacterial products for everyday care. A group of researchers, artists and hackers have created a platform to engage in citizen’s science projects such as public workshops and networking events with the purpose to facilitate the understanding of scientific research in the field of biotechnologies, but also to develop open-source and DIY/DIWO tools [92]. By turning to alternative models of knowledge sharing, members of the “Hackteria” collective refuse to distinguish between artistic production and scientific research, affirming the possibility of other solutions that are not heavily dependent from financial support, and consequently from the endorsement of funding agencies and commercial partners, from which both the scientific and the artistic fields strongly depend. The power of synthetic biology is uncovered by the work of Alexandra Daisy Ginsberg “Designing for the Sixth Extinction” [93]. In her science-fiction-oriented scenarios, synthetic species counterintuitively provide a tool for bioremediation and preservation of biological diversity. Here, scientific concepts and artistic creativity merge for a shared endeavour [74].

Artists should not simply act as translators of scientific ideas: they can shape them as well as the directions of the scientific research into areas that are more relevant and urgent for our society. With the words of the recently departed chemistry Nobel Prize laureate and graphic artist Harry Kroto: “In science, the universe is in control; in art, you are” [94]. The perspective is in either case both scaring and fascinating, the outcomes—much in good science as in good art—unpredictable and enlightening.

## A last word

It is essential that society engages in the debate on the importance of science in general, and in particular on those of microorganisms and their biotechnological potential. However, to counteract the antibiotic crisis, and to inform about the urgent necessity of this, is not an easy task. We believe that insights about communication *among* microorganisms—gained by microbiologists with appropriate new tools and approaches—as well as *on* microorganisms—by the concerted action of scientists, communicators, artists, scientific institutes and the general public—are acutely needed.

As the chemist, entrepreneur and pioneer in microbiology and immunology Louis Pasteur is often quoted with: “Messieurs, c’est les microbes qui auront le dernier mot!” (“Gentlemen, it’s the microbes who will have the last word!”). If we uncover microbial interactions and communication to the fullest, and talk more about microorganisms, there are good chances it is going to be a nice word.
